# Role of imitation and limited rehabilitation capacity on the spread of drug abuse

**DOI:** 10.1186/s13104-018-3574-4

**Published:** 2018-07-18

**Authors:** Josiah Mushanyu

**Affiliations:** Department of Mathematics, University of Zimbabwe, Box MP 167, Mount Pleasant, Harare, Zimbabwe

**Keywords:** Drug abuse, Imitation, Reproduction number, Rehabilitation capacity

## Abstract

**Objectives:**

We formulate a mathematical model for the spread of drug abuse using non linear ordinary differential equations. The model seeks to investigate both peer influence and limited rehabilitation effects on the dynamics of drug abuse. Peer-influence is modelled through the mechanism of imitation and limited rehabilitation is described using a special treatment function. Center manifold theory is used to show that the model exhibits the phenomenon of backward bifurcation. Matlab has been used to carry out numerical simulations to support theoretical findings.

**Results:**

The model analysis shows that the model has multiple equilibria. It has been shown that the classical $\mathcal {R}_a$—threshold is not the key to control drug abuse within a population. In fact drug abuse problems may persist in the population even with subthreshold values of $\mathcal {R}_a$. This was shown to result, in particular when, $\omega$, $\eta _1$ and $\eta _2$ are high enough such that $\omega >\omega ^*$, $\eta _1>\eta ^*_1$ and $\eta _2>\eta ^*_2$. The results suggest the need for comprehensive and accessible substance abuse treatment services to curtail drug abuse.

**Electronic supplementary material:**

The online version of this article (10.1186/s13104-018-3574-4) contains supplementary material, which is available to authorized users.

## Introduction

Drug abuse has increased in recent years and is now an epidemic globally. The magnitude of the world drug problem becomes more apparent when considering that more than 1 out of 10 drug users is a problem drug user and the vast majority of these individuals continue to have no access to treatment [1]. There continues to be a large “treatment gap” for substance abuse problems as many countries have a large shortfall in the provision of services. According to the United Nations Office on Drugs and Crime [1], only one out of every six problem drug users in the world has access to treatment. Generally, the number of patients in need of rehabilitation often exceeds the carrying capacities of drug treatment facilities, especially those funded by the state.

Several mathematical models describing the spread of psycho-social ills in a community have been proposed, see for example, drug epidemics [2–9], alcoholism [10–16], smoking [17–19]. The basic assumption in most drug abuse models is that there is a direct proportional relationship between the number of drug users in need of treatment and the available health care resources present. In this paper, we develop a mathematical model that takes into account the possibility of the number of drug abusers in need of rehabilitation exceeding the capacity of rehabilitation centers. Recruitment into rehabilitation (inpatient or outpatient) is denoted by *H*(*U*) and defined as follows:

1 $$\begin{aligned} H(U)=\frac{\alpha U}{1+\omega U} \end{aligned}$$

where *U* represents the proportion of individuals abusing drugs, $\alpha$ is the maximum rehabilitation uptake per unit of time and $\omega$ measures the extent of the effect of the problem of demand for treatment. Firstly, observe that for small *U*, $H(U)\approx \alpha U$, that is, when the number of drug users is not too large, then the rate of entering treatment is proportional to the number of drug users present. Secondly, observe that for large *U*, $H(U)\approx \alpha /\omega$, this means that the rate of entering rehabilitation takes a maximum value $\alpha /\omega$. Finally, when $\omega =0$, we again obtain the result as in the first case, $H(U)=\alpha U$, that is, the function returns to a linear function mostly used in previous drug abuse models. Amongst drug abusers who are seeking help through rehabilitation, we have that a proportion *p* of these individuals are recruited into inpatient rehabilitation and the complementary proportion $(1-p)$ are recruited into outpatient rehabilitation. It is also important to note that epidemic models including treatment functions of the form (1) are found in [20–23].

We also include peer influence effects on the spread of drug abuse by assuming that the recruitment process happens through the mechanism of imitation. In this paper, we use the recruitment function given in [11]. Compared to previous drug epidemic models [2–9], a key novelty of our model is the inclusion of both imitation and limited rehabilitation on the dynamics of drug abuse.

The paper is arranged as follows; in “Model formulation” section, we formulate and establish the basic properties of the model. The model is analysed for stability in “Model analysis” section. In “Numerical simulations” section, we carry out some numerical simulations. Parameter estimation is also presented in this section. The paper is concluded in the “Conclusions” section.

## Main text

### Model formulation

The model divides the population into four classes, *S*(*t*), *U*(*t*), $R_{op}(t)$ and $R_{ip}(t)$. The class *S*(*t*) represents the population at risk of being initiated into drug abuse. The class *U*(*t*) denotes those abusing drugs, $R_{op}(t)$ denotes those in rehabilitation as out-patients and $R_{ip}(t)$ denotes those in rehabilitation as in-patients. The total local population is thus given by

$$\begin{aligned} N(t)=S(t)+U(t)+R_{op}(t)+R_{ip}(t). \end{aligned}$$

The general population enter the susceptible population at a rate $\Lambda$, that is, the demographic process of individuals reaching age 15 in the modelling time period. Susceptible individuals become drug users upon contact with individuals in compartments *U* or $R_{op}$. This results from the assumption that those in inpatient rehabilitation do not have contact with the population at risk. The per capita contact rate $\beta _1$ is a product of the effective number of contacts $c_1$, between drug users not in rehabilitation and the susceptible population, and the probability $\hat{\beta _1}$, that a contact results into initiation into drug use, that is, $\beta _1=c_1\hat{\beta _1}$. The per capita contact rate $\beta _2$ is a product of the effective number of contacts $c_2$, between drug users in outpatient rehabilitation and the susceptible population, and the probability $\hat{\beta _2}$, that a contact results into initiation into drug use, that is, $\beta _2=c_2\hat{\beta _2}$. Individuals under outpatient rehabilitation quit drug abuse permanently at a rate $\delta _1$ and individuals under inpatient rehabilitation quit drug abuse permanently at a rate $\delta _2$. The general population experience natural death at a rate $\mu$. Drug users undergoing outpatient rehabilitation relapse into drug use at a rate $\rho _1$ whereas those undergoing inpatient rehabilitation relapse at a rate $\rho _2$. The relapse is thus assumed to be a voluntary process, that is not influenced by interaction with users. We allow the transfer from outpatient to inpatient rehabilitation, this happens at a rate $\gamma _1$. We also allow the transfer from inpatient to outpatient rehabilitation, this rate is represented by $\gamma _2$. We assume that individuals in each compartment are indistinguishable and there is homogeneous mixing. We have the following general set of nonlinear ordinary differential equations:

2 $$\begin{aligned} \left\{ \begin{array}{llll} \dfrac{dS}{dt}&{}=&{}\Lambda -f(S,U,R_{op})-\mu S, \\ \dfrac{dU}{dt}&{}=&{}f(S,U,R_{op})+\rho _1 R_{op}+\rho _2 R_{ip}-\mu U-\dfrac{\alpha U}{1+\omega U},\\ \dfrac{dR_{op}}{dt}&{}=&{}\gamma _2 R_{ip}-(\mu +\gamma _1+\rho _1+\delta _1)R_{op}+\dfrac{(1-p)\alpha U}{1+\omega U},\\ \dfrac{dR_{ip}}{dt}&{}=&{}\gamma _1 R_{op}-(\mu +\gamma _2+\rho _2+\delta _2)R_{ip}+\dfrac{p\alpha U}{1+\omega U}, \end{array} \right. \end{aligned}$$

with the initial conditions:

$$\begin{aligned} S(0)= & {} S_0>0,~U(0)=U_0\ge 0,~R_{op}(0)=R_{op0}\ge 0,~R_{ip}(0)=R_{ip0}\ge 0, \end{aligned}$$

where

$$\begin{aligned} f(S,U,R_{op})= & \; \beta _1 SU(1+\eta _1 U)+\beta _2 SR_{op} (1+\eta _2 R_{op})\\= & \; \beta _1 \left( SU(1+\eta _1 U)+\theta SR_{op} (1+\eta _2 R_{op})\right) . \end{aligned}$$

Here $\beta _2=\theta \beta _1$, with $\theta =1$ signifying that the chance of initiating drug abuse habit upon contact with an individual in *U* or $R_{op}$ is the same, $\theta \in (0,1)$ signifying a reduced chance of initiating drug abuse habit upon contact with an individual in $R_{op}$ as compared to an individual in *U*, $\theta >1$ signifies an increased rate of initiating drug abuse habit upon contact with an individual in $R_{op}$ as compared to an individual in *U*.

### Model analysis

#### Model properties

##### Invariant region

It follows from system (2) that

3 $$\begin{aligned} \frac{dN}{dt}\le \Lambda -\mu (S+U+R_{op}+R_{ip}). \end{aligned}$$

Then, $\displaystyle \limsup _{t\rightarrow \infty } N\le \dfrac{\Lambda }{\mu }$. Thus, the feasible region for system (2) is

4 $$\begin{aligned} \Omega =\left\{ (S,U,R_{op},R_{ip})\in \mathbb {R}^{4}_{+}|~N\le \frac{\Lambda }{\mu }\right\} . \end{aligned}$$

It is easy to verify that the region $\Omega$ is positively invariant with respect to system (2), see for instance [3–5].

### The drug-free equilibrium and the abuse reproduction number

Model system (2) always has a drug-free equilibrium $\mathcal {D}_0=\left( \dfrac{\Lambda }{\mu },\ 0,\ 0,\ 0\right)$. Denote the abuse reproduction number of model system (2) by

$$\begin{aligned} \mathcal {R}_a = & \; \mathcal {R}_{U}+\mathcal {R}_{R_{op}}~~~\text{ where }~~~\\ \mathcal {R}_{U}= & \; \left( \dfrac{\Lambda }{\mu }\right) \left[ \frac{\beta _1 (1-\Phi _1)}{\mu (1-\Phi _1)+\alpha p(1-\Phi _2) +\alpha (1-p)(1-\Phi _3)}\right] ~~~\text{ and }~~~\\ \mathcal {R}_{R_{op}}= & \; \left( \frac{\Lambda }{\mu h_1h_2}\right) \left[ \frac{\beta _2 \left((1-p)\alpha h_2+p\alpha \gamma _2 \right)}{\mu (1-\Phi _1)+\alpha p(1-\Phi _2) +\alpha (1-p)(1-\Phi _3)}\right] \end{aligned}$$

with

$$\begin{aligned} \Phi _1= & \; \frac{\gamma _1\gamma _2}{h_1h_2},~\Phi _2=\frac{\gamma _1\gamma _2 +\gamma _2\rho _1+\rho _2 h_1}{h_1h_2},~\Phi _3=\frac{\gamma _1\gamma _2 +\gamma _1\rho _2 +\rho _1 h_2}{h_1h_2},\\ h_1= & \; \mu +\gamma _1+\rho _1+\delta _1~~\text{ and }~~h_2=\mu +\gamma _2+\rho _2+\delta _2. \end{aligned}$$

We can clearly note that $\gamma _1\gamma _2\le h_1h_2$ and so $(1-\Gamma _1)\ge 0$. Also, $\gamma _1\gamma _2 +\gamma _2\rho _1+\rho _2 h_1\le h_1h_2$ and $\gamma _1\gamma _2 +\gamma _1\rho _2 +\rho _1 h_2\le h_1h_2$. Therefore, $\mathcal {R}_a$ is non-negative. The abuse reproduction number $\mathcal {R}_a$ of the model, is the average number of secondary cases generated by one drug user during his/her duration of drug use in a population of completely potential drug users.

### Local stability of the drug-free steady state

#### **Theorem 1**

*The drug-free equilibrium* $\mathcal {D}_0$* is locally asymptotically stable when* $\mathcal {R}_a<1$* and is unstable when* $\mathcal {R}_a>1$.

#### *Proof*

The Jacobian matrix of model system Eq. (2) at $\mathcal {D}_0$ is given by

$$\begin{aligned} J(\mathcal {D}_0)=\begin{bmatrix} -\mu&-\frac{\Lambda }{\mu }\beta _1&\frac{\Lambda }{\mu }\beta _2&0\\ 0&g_1&g_2&\rho _2\\ 0&(1-p)\alpha&-h_1&\gamma _2\\ 0&p\alpha&\gamma _1&-h_2 \end{bmatrix} \end{aligned}$$

where $h_1$ and $h_2$ are defined as before and $g_1=\frac{\Lambda }{\mu }\beta _1-(\mu +\alpha )$, $g_2=\frac{\Lambda }{\mu }\beta _2+\rho _1$. The local stability of the drug-free equilibrium is determined by the following submatrix of $J(\mathcal {D}_0)$,

$$\begin{aligned} \bar{J}(\mathcal {D}_0)=\begin{bmatrix} g_1&g_2&\rho _2\\ (1-p)\alpha&-h_1&\gamma _2\\ p\alpha&\gamma _1&-h_2 \end{bmatrix}. \end{aligned}$$

Since all off-diagonal elements are positive, we now consider matrix $-\bar{J}(\mathcal {D}_0)$. We claim that $-\bar{J}(\mathcal {D}_0)$ is an* M*—matrix. Multiplying matrix $-\bar{J}(\mathcal {D}_0)$ by the positive $3\times 1$ matrix

$$\begin{aligned} W_1=\begin{bmatrix} h_1h_2(1-\Phi _1)\\\ p\alpha \gamma _2+(1-p)\alpha h_2\\ (1-p)\alpha \gamma _1 + p\alpha h_1 \end{bmatrix}, \end{aligned}$$

we have

$$\begin{aligned} -\bar{J}(\mathcal {D}_0)\cdot W_1=(1-\mathcal {R}_a)\cdot W_2 \end{aligned}$$

where $W_2$ is a positive $3\times 1$ matrix given by

$$\begin{aligned} W_2=\begin{bmatrix} h_1h_2\left[ \mu (1-\Phi _1)+\alpha p(1-\Phi _2)+\alpha (1-p)(1-\Phi _3)\right] \\ 0\\ 0 \end{bmatrix}. \end{aligned}$$

Then, it follows from* M*—matrix theory that all eigenvalues of $\bar{J}(\mathcal {D}_0)$ have negative real parts, which implies the local asymptotic stability of the drug-free equilibrium if $\mathcal {R}_a<1$. On the other hand, it can be shown that the determinant of $\bar{J}(\mathcal {D}_0)$ is given by

$$\begin{aligned} \text{ det }~\bar{J}(\mathcal {D}_0)=h_1h_2\left[ \mu (1-\Phi _1)+\alpha p(1-\Phi _2)+\alpha (1-p)(1-\Phi _3)\right] (\mathcal {R}_a-1). \end{aligned}$$

Thus, if $\mathcal {R}_a<1$, then matrix $\bar{J}(\mathcal {D}_0)$ has eigenvalues with negative real parts, which implies the stability of the drug-free equilibrium. This completes the proof.$\square$

### The drug-persistent equilibrium point

The drug-persistent equilibrium $\mathcal {D}^*=\left( S^*,U^*,R^*_{op},\;R^*_{ip}\right)$ always satisfies

5 $$\begin{aligned} \left\{ \begin{array}{llll} &{}&{}\Lambda -f\left( S^*,U^*,R^*_{op}\right) -\mu S^*=0, \\ &{}&{}f\left( S^*,U^*,R^*_{op}\right) +\rho _1 R^*_{op}+\rho _2 R^*_{ip}-\mu U^*-\dfrac{\alpha U^*}{1+\omega U^*}=0,\\ &{}&{}\gamma _2 R^*_{ip}-(\mu +\gamma _1+\rho _1+\delta _1)R^*_{op}+\dfrac{(1-p)\alpha U^*}{1+ \omega U^*}=0,\\ &{}&{}\gamma _1 R^*_{op}-(\mu +\gamma _2+\rho _2+\delta _2)R^*_{ip}+\dfrac{p\alpha U^*}{1+ \omega U^*}=0. \end{array} \right. \end{aligned}$$

From the last two equations of (5) we have that

6 $$\begin{aligned} R^*_{op}=\frac{\Psi _1 U^*}{1+\omega U^*}~~\text{ and }~~R^*_{ip}=\frac{\Psi _2 U^*}{1+\omega U^*} \end{aligned}$$

where

$$\begin{aligned} \Psi _1=\frac{\alpha p \gamma _2+\alpha (1-p)h_2}{h_1h_2\left( 1-\Phi _1\right) }~~\text{ and }~~\Psi _2=\frac{\alpha p h_1+\alpha (1-p)\gamma _1}{h_1h_2\left( 1-\Phi _1\right) }. \end{aligned}$$

Substituting expressions (6) into the first equation of (5), we get

7 $$\begin{aligned} S^*=\frac{\Lambda \left( 1+\omega U^*\right) ^2}{\left( \mu +\beta _1 U^*(1+\eta _1 U^*)\right) (1+\omega U^*)^2 +\beta _2 \Psi _1 U^*\left( 1+\omega U^* +\eta _2\Psi _1 U^*\right) }. \end{aligned}$$

Substituting expressions (6) and (7) into the second equation of (5) leads to the following sixth order polynomial equation

8 $$\begin{aligned} U^*\left( \chi _5 U^{*5}+\chi _4 U^{*4}+\chi _3 U^{*3}+\chi _2 U^{*2}+\chi _1 U^* +\chi _0\right) =0. \end{aligned}$$

Solving (8) gives $U^*=0$ which corresponds to the drug-free equilibrium or

9 $$\begin{aligned} \chi _5 U^{*5}+\chi _4 U^{*4}+\chi _3 U^{*3}+\chi _2 U^{*2}+\chi _1 U^* +\chi _0=0, \end{aligned}$$

where the coefficients $\chi _i,~1\le i\le 5$ are in Additional file 1: Appendix S1. We can clearly note that, $\chi _0>0\Leftrightarrow \mathcal {R}_a<1$ and $\chi _0<0\Leftrightarrow \mathcal {R}_a>1$. The number of possible positive real roots of the polynomial (9) can be determined using the Descartes Rule of Signs. The number of positive roots are at most five.

### Backward bifurcation

Conditions for the existence of backward bifurcation follow from Theorem 4.1 proven in [24]. Let us make the following change of variables:

$S=x_{1},~U=x_2~R_{op}=x_3,~R_{ip}=x_4$, so that $\text{ N }=\displaystyle \sum _{n=1}^{4}{x_n}$. We now use the vector notation $X=(x_{1},x_{2},x_{3},x_{4})^{T}$. System (2) can be written in the form $\dfrac{dX}{dt}=F(t,x(t))=(f_{1},f_{2},f_{3},f_{4})^T$, where

10 $$\begin{aligned} \left\{ \begin{array}{llll} x^{'}_{1}(t)&{}=&{}p\Lambda -h(x_1,x_2,x_3)-\mu x_1=f_1,\\ x^{'}_{2}(t)&{}=&{}h(x_1,x_2,x_3)+\rho _1 x_3 +\rho _2 x_4 -\mu x_2-\dfrac{\alpha x_2}{1+ \omega x_2}=f_{2},\\ x^{'}_{3}(t)&{}=&{}\gamma _2 x_4-h_1 x_3 +\dfrac{\alpha (1-p)x_2}{1+\omega x_2}=f_{3},\\ x^{'}_{4}(t)&{}=&{}\gamma _1 x_3-h_2 x_4 +\dfrac{\alpha p x_2}{1+\omega x_2}=f_{4}, \end{array} \right. \end{aligned}$$

with

$$\begin{aligned} h(x_1,x_2,x_3)=\beta _1\left( x_1x_2(1+\eta _1 x_2)+\theta x_1x_3(1+\eta _2 x_3)\right) . \end{aligned}$$

Let $\beta _1$ be the bifurcation parameter, $\mathcal {R}_a=1$ corresponds to

11 $$\begin{aligned} \beta _1=\beta ^*_1=\left( \frac{\mu }{\Lambda }\right) \left[ \frac{h_1h_2(\mu (1-\Phi _1)+\alpha p(1-\Phi _2) +\alpha (1-p)(1-\Phi _3))}{h_1h_2(1-\Phi _1)+\alpha p \theta \gamma _2 +\alpha (1-p)\theta h_2}\right] . \end{aligned}$$

The Jacobian matrix of system (2) at $\mathcal {D}_0$ when $\beta _1=\beta ^*_1$ is given by

$$\begin{aligned} J^*(\mathcal {D}_0)=\begin{bmatrix} -\mu&-\frac{\Lambda }{\mu }\beta ^*_1&\frac{\Lambda }{\mu }\theta \beta ^*_1&0\\\\ 0&g^*_1&g^*_2&\rho _2\\ 0&(1-p)\alpha&-h_1&\gamma _2\\ 0&p\alpha&\gamma _1&-h_2 \end{bmatrix} \end{aligned}$$

where $h_1$ and $h_2$ are defined as before and $g^*_1=\frac{\Lambda }{\mu }\beta ^*_1-(\mu +\alpha )$, $g^*_2=\frac{\Lambda }{\mu }\theta \beta ^*_1+\rho _1$.

System (10), with $\beta _1=\beta ^*_1$ has a simple eigenvalue, hence the center manifold theory can be used to analyse the dynamics of system (2) near $\beta _1=\beta ^*_1$. It can be shown that $J^*(\mathcal {D}_0)$, has a right eigenvector given by $w=(w_1,w_2,w_3,w_4)^{T}$, where

$$\begin{aligned} w_1= & \; -h_1h_2 \left(\mu (1-\Phi _1)+\alpha p (1-\Phi _2)+\alpha (1-p)(1-\Phi _3)\right),\\ w_2= & \; \mu h_1h_2(1-\Phi _1),~~w_3=\alpha \mu ((1-p)h_2+p\gamma _2),~~w_4=\alpha \mu (p h_1+(1-p)\gamma _1). \end{aligned}$$

Further, the left eigenvector of $J^*(\mathcal {D}_0)$, associated with the zero eigenvalue at $\beta _1=\beta ^*_1$ is given by $v=(v_1,v_2,v_3,v_4)^{T}$, where

$$\begin{aligned} v_1= & \; 0,~~v_2=h_1h_2\left( 1-\Phi _1\right) +\alpha (1-p)\theta h_2 +\alpha p\theta \gamma _2,\\ v_3= & \; h_2 \left( \theta (\alpha +\mu )+ \rho _1\right) +\rho _2 \left( \gamma _1 -\alpha \theta p\right) ,\\ v_4= & \; \rho _2 \left( h_1 +\alpha \theta (1-p)\right) +\gamma _2 \left( \theta (\alpha + \mu )+\rho _1\right) . \end{aligned}$$

The computations of **a** and **b** are necessary in order to apply Theorem 4.1 in Castillo-Chavez and Song [24]. For system (10), the associated non-zero partial derivatives of *F* at the drug-free equilibrium are in Additional file 1: Appendix S2. It thus follows that

$$\begin{aligned} \text{\bf{a}}= & \; v_1w_1w_2\frac{\partial ^2 f_1}{\partial x_1\partial x_2}+v_1w_1w_3\frac{\partial ^2 f_1}{\partial x_1\partial x_3}+v_1w^2_2\frac{\partial ^2 f_1}{\partial x^2_2}+v_1w^2_3\frac{\partial ^2 f_1}{\partial x^2_3}+v_2w_1w_2\frac{\partial ^2 f_2}{\partial x_1\partial x_2}\\&+v_2w_1w_3\frac{\partial ^2 f_2}{\partial x_1\partial x_3}+v_2w^2_2\frac{\partial ^2 f_2}{\partial x^2_2}+v_2w^2_3\frac{\partial ^2 f_2}{\partial x^2_3}+v_3w^2_2\frac{\partial ^2 f_3}{\partial x^2_2}+v_4w^2_2\frac{\partial ^2 f_4}{\partial x^2_2}\\= & \; 2\alpha \omega v_2w^2_2-2(1-p)\alpha \omega v_3 w^2_2 -2\alpha p\omega v_4w^2_2+\beta ^*_1v_2w_1w_2+\theta \beta ^*_1 v_2w_1w_3\\&+\frac{2\Lambda \beta ^*_1\eta _1 v_2w^2_2}{\mu }+\frac{2\theta \Lambda \beta ^*_1\eta _2 v_2 w^2_3}{\mu }\\= & \; \left[ A\omega -\mu ^2h_1h_2(1-\Phi _1)v^2_2\beta ^*_1\right] +\left[ B\eta _1-\mu \alpha p h_1h_2(1-\Phi _2)v^2_2\beta ^*_1\right] \\&+ \left[ C\eta _2 -\mu \alpha (1-p)h_1h_2(1-\Phi _3)v^2_2\beta ^*_1\right] \\= & {} A\left( \omega -\omega ^*\right) +B\left( \eta _1 -\eta ^*_1\right) +C\left( \eta _2 - \eta ^*_2\right) , \end{aligned}$$

where

12 $$\begin{aligned} \omega ^*=\frac{\mu ^2h_1h_2(1-\Phi _1)v^2_2\beta ^*_1}{A},~~\eta ^*_1=\frac{\mu \alpha p h_1h_2(1-\Phi _2)v^2_2\beta ^*_1}{B},~~\eta ^*_2=\frac{\mu \alpha (1-p)h_1h_2(1-\Phi _3)v^2_2\beta ^*_1}{C}, \end{aligned}$$

with

$$\begin{aligned} A= & \; 2\alpha \mu ^2h^2_1h^2_2\left( 1-\Phi _1\right) ^2\times \left[ ((1-\theta )\mu +\delta _1)((1-p)\rho _2+\gamma _2)+(\mu +\delta _2)((1-\theta )\mu +\mu \theta p+\gamma _1+\delta _1 +p\rho _1)\right] ,\\ B= & \; 2\Lambda \mu h^2_1h^2_2\left( 1-\Phi _1\right) ^2v_2\beta ^*_1~~\text{ and }~~C=2\Lambda \mu \theta \alpha ^2((1-p)h_2+p\gamma _2)^2v_2\beta ^*_1. \end{aligned}$$

Note that $\omega ^*>0$, $\eta ^*_1>0$ and $\eta ^*_2>0$. Also note that if $\omega >\omega ^*$, $\eta _1>\eta ^*_1$ and $\eta _2>\eta ^*_2$ then $\text{\bf{a}}>0$ and $\text{\bf{a}}<0$ if $\omega <\omega ^*$, $\eta _1<\eta ^*_1$ and $\eta _2<\eta ^*_2$. Lastly,

$$\begin{aligned} \text{\bf{b}}=\Lambda \left( h_2 \left( \alpha \theta (p-1)-h_1\right) +\gamma _2 \left( \gamma _1-\alpha \theta p\right) \right) {}^2>0. \end{aligned}$$

We thus have the following result

#### **Theorem 2**

If $\omega >\omega ^*$, $\eta _1>\eta ^*_1$ and $\eta _2>\eta ^*_2$, then model system (2) has a backward bifurcation at $\mathcal {R}_a=1$.

### Results and discussion

#### Numerical simulations

##### Parameter estimation

Since we can rarely enumerate the incidence of drug users, data from treatment centers can be used as proxy for estimating parameters for drug related issues. We use data obtained from previous mathematical models with inpatient and outpatient rehabilitation [4, 5]. Some of the parameter values will be obtained from literature.

**Table 1:** Parameter values used in numerical simulations

Parameter	Range	Value	Source
$\beta _1$	0.10–0.21	0.105	[7]
$\beta _2$	0–0.10	0.063	[6]
$\omega$	0–1	0.62	[5]
$\alpha$	0–0.05024	0.02827	[4]
*p*	0–1	0.352	[4]
$\eta _1$	0–1	0.24	Assumed
$\eta _2$	0–1	0.13	Assumed
$\delta _1$	0.001–1	0.01	[4]
$\delta _2$	0.01–1	0.3142	[4]
$\rho _1$	0–0.054	0.0382	[4]
$\rho _2$	0–0.0235	0.0020	[4]
$\gamma _1$	0–0.06012	0.02961	[4]
$\gamma _2$	0–0.008	0.003	[4]
$\Lambda$	0.028–0.080	0.04	[7]
$\mu$	0.019–0.021	0.020	[25]

Parameter values used for numerical simulations are given in Table 1.

##### Numerical results

We carry out detailed numerical simulations using matlab to support our theoretical findings. The initial conditions used are: $S(0)=0.95$, $U(0)=0.05$, $R_{op}(0)=0$, $R_{ip}(0)=0$.

**Fig. 1:**
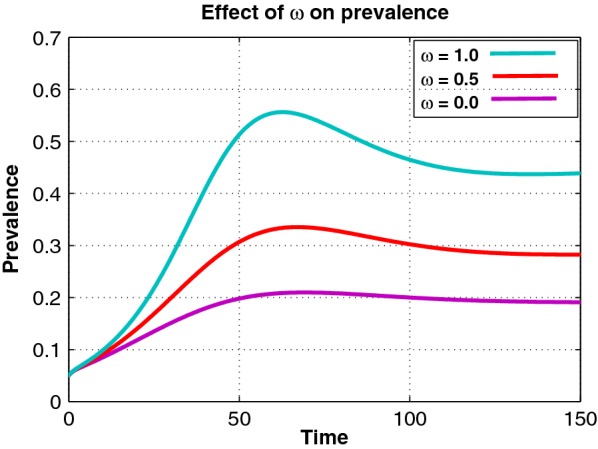
Effects of varying $\omega$ on the prevalence of drug abuse, starting from 0 up to 1.0 with a step size of 0.5

**Fig. 2:**
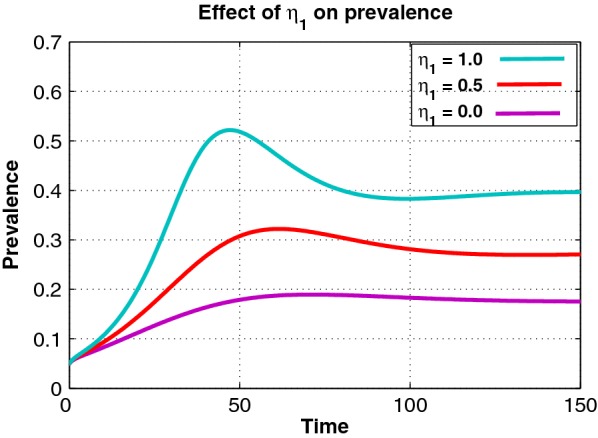
Effects of varying $\eta _1$ on the prevalence of drug abuse, starting from 0 up to 1.0 with a step size of 0.5

Figures 1 and 2 illustrate the effect of varying parameters $\omega$ and $\eta _1$ on the prevalence of drug abuse. Figures 1 and 2 demonstrate that increasing $\omega$ and $\eta _1$ results in an increase in the prevalence of drug abuse. This is a reflection that limited rehabilitation and imitation are of major concern in the fight against drug abuse.

## Conclusions

A mathematical model that incorporates imitation and limited rehabilitation has been formulated using nonlinear ordinary differential equations. It has been shown that the classical $\mathcal {R}_a$—threshold is not the key to control drug abuse within a population. In fact drug abuse problems may persist in the population even with subthreshold values of $\mathcal {R}_a$. This was shown to result, in particular when $\omega$, $\eta _1$ and $\eta _2$ are high enough such that $\omega >\omega ^*$, $\eta _1>\eta ^*_1$ and $\eta _2>\eta ^*_2$. Considerable effort should be directed towards reducing $\omega$, $\eta _1$ and $\eta _2$, this done by increasing the value of $\omega ^*$, $\eta ^*_1$ and $\eta ^*_2$ so as to avoid backward bifurcation. Also, results from the model application show that increasing $\omega$ and $\eta _1$ lead to an increase in the prevalence of drug abuse. Thus, communities should have suitable capacity for the treatment of drug abusers and specific health and/or education programs may be employed to reduce the imitation coefficient $\eta _1$.

## Limitations

Like in any model development, the model is not without limitations.

- The model did not take into account contextual dynamics, such as drug supply chains or changes in interdiction.
- Also, the study presented here ignored detailed social and economic characteristics.
- Other initiation processes, not included in this work, for instance, initiation by self-conversion, drug supply chains etc. may form part of the author’s future research considerations.

## Additional file

**Additional file 1.** Appendices S1, S2.
