# Supplementary material for: Role of imitation and limited rehabilitation capacity on the spread of drug abuse
Source: BMC Res Notes. 2018 Jul 18;11:493. doi: 10.1186/s13104-018-3574-4 (PMC6052710; doi:10.1186/s13104-018-3574-4)
Supplement: Supplementary file 1 — Additional file 1. Appendices S1, S2. [file 13104_2018_3574_MOESM1_ESM.pdf]

Appendix S1. Coefficients of polynomial (9)

$$\begin{aligned}
\chi_0 &= \frac{\mu}{(1-\Phi_1)}\left[\mu(1-\Phi_1)-\alpha p(1-\Phi_2)+\alpha(1-p)(1-\Phi_3)\right](1-\mathcal{R}_a),\\
\chi_1 &= \beta_1(\alpha-\eta_1\Lambda-3\Lambda\omega+\mu-\rho_1\Psi_1-\rho_2\Psi_2)+\mu\omega(2\alpha+3\mu-2\rho_1\Psi_1-2\rho_2\Psi_2)\\
&\quad+\beta_2\Psi_1(\alpha-\Psi_1(\eta_2\Lambda+\rho_1)-2\Lambda\omega+\mu-\rho_2\Psi_2),\\
\chi_2 &= \beta_1(\eta_1(\alpha-3\Lambda\omega+\mu-\rho_1\Psi_1-\rho_2\Psi_2)+\omega(2\alpha-3\Lambda\omega+3\mu-2\rho_1\Psi_1-2\rho_2\Psi_2))\\
&\quad-\beta_2\Psi_1(\eta_2\Psi_1(-\alpha+\Lambda\omega-\mu+\rho_1\Psi_1+\rho_2\Psi_2)+\omega(-\alpha+\Lambda\omega-2\mu+\rho_1\Psi_1+\rho_2\Psi_2))\\
&\quad+\mu\omega^2(\alpha+3\mu-\rho_1\Psi_1-\rho_2\Psi_2),\\
\chi_3 &= \omega\beta_1(\eta_1(2\alpha-3\Lambda\omega+3\mu-2\rho_1\Psi_1-2\rho_2\Psi_2)-\omega(-\alpha+\Lambda\omega-3\mu+\rho_1\Psi_1+\rho_2\Psi_2))\\
&\quad+\omega\mu\left(\beta_2\Psi_1(\eta_2\Psi_1+\omega)+\mu\omega^2\right),\\
\chi_4 &= \beta_1\omega^2(\eta_1(\alpha-\Lambda\omega+3\mu-\rho_1\Psi_1-\rho_2\Psi_2)+\mu\omega),\\
\chi_5 &= \beta_1\eta_1\mu\omega^3.
\end{aligned}$$

Appendix S2. Associated non-zero partial derivatives of F at the drug-free equilibrium

$$\begin{aligned}
\frac{\partial^2 f_1}{\partial x_1\partial x_2} &= \frac{\partial^2 f_1}{\partial x_2\partial x_1}=-\beta_1^*,\quad \frac{\partial^2 f_1}{\partial x_1\partial x_3}=\frac{\partial^2 f_1}{\partial x_3\partial x_1}=-\theta\beta_1^*,\\
\frac{\partial^2 f_1}{\partial x_2^2} &= \frac{-2\Lambda\beta_1^*\eta_1}{\mu},\quad \frac{\partial^2 f_1}{\partial x_3^2}=\frac{-2\Lambda\theta\beta_1^*\eta_2}{\mu},\\
\frac{\partial^2 f_2}{\partial x_1\partial x_2} &= \frac{\partial^2 f_2}{\partial x_2\partial x_1}=\beta_1^*,\quad \frac{\partial^2 f_2}{\partial x_1\partial x_3}=\frac{\partial^2 f_2}{\partial x_3\partial x_1}=\theta\beta_1^*,\\
\frac{\partial^2 f_2}{\partial x_2^2} &= 2\alpha\omega+\frac{2\Lambda\beta_1^*\eta_1}{\mu},\quad \frac{\partial^2 f_2}{\partial x_3^2}=\frac{2\Lambda\theta\beta_1^*\eta_2}{\mu},\\
\frac{\partial^2 f_3}{\partial x_2^2} &= -2(1-p)\alpha\omega,\quad \frac{\partial^2 f_4}{\partial x_2^2}=-2p\alpha\omega,\\
\frac{\partial^2 f_1}{\partial x_2\partial\beta_1^*} &= \frac{-\Lambda}{\mu},\quad \frac{\partial^2 f_1}{\partial x_3\partial\beta_1^*}=\frac{-\theta\Lambda}{\mu},\\
\frac{\partial^2 f_2}{\partial x_2\partial\beta_1^*} &= \frac{\Lambda}{\mu},\quad \frac{\partial^2 f_2}{\partial x_3\partial\beta_1^*}=\frac{\theta\Lambda}{\mu}.
\end{aligned}$$
